# Woman Workers' World

**Published:** 1919-10-11

**Authors:** 


### Woman Workers' World.

Light sweets for the convalescent used to be made most attractive by the addition of a little—even ever so little—whipped cream. The white-of-egg substitutes are rather an irritating kind of fraud, but the following American recipe has much charm, and is nourishing:—1 can evaporated milk, 1 teaspoon gelatine, ½ teaspoon vanilla, 1 tablespoon sugar, 1 tablespoon boiling water. Soak gelatine in 2 teaspoons cold water for 5 minutes. Dissolve in boiling water, add to milk. Beat for a minute and chill. Then beat till thick. Add vanilla and sugar.

From America comes also the story of a young housekeeper who complained of the small eggs offered her. The grocer replied that those were what the farmer had just sent. "Yes," she replied, and explained that the farmers were profiteering scandalously. "They are so anxious to get their eggs sold that they take them off the nest far too soon."

An interesting experiment is shortly to be tried by the Young Women's Christian Association to enable some at least of those working girls who become deeply interested in study through evening classes or continuation schools to satisfy the hunger of their minds. A house is to be taken, probably in the neighbourhood of London or Cambridge, where the simple life may be lived for a year at a cost of £60 and a year's college life be gained. From such a College for Working Girls it is hoped that students would go out to uplift the life of their own occupation and surroundings, not merely to aim at climbing a social ladder. It may be hoped, too, that here the beginnings of a new altruism would be found. Bursaries and scholarships given by societies or groups of people are planned and hoped for.

Two facts of interest to nurses come from the British Women's Emigration Association. The Victorian Order of Nurses at Ottawa can at present accept and use any number of trained nurses with C.M.B. diploma. Nurses wishing to serve in Australia and fully trained can become members of the Australian Association of Trained Nurses, and then will get good posts easily.

Judging by the present correspondence on the subject, women are determined that parlours shall be provided when the new houses are built. Ealing Women Citizens' Association are extremely indignant that as regards the 230 houses and flats approved by Dr. Addison, only 25 per cent. are to be provided with parlours. When there is a family the parlour acts as a study, and with a prospect of young people being at school until eighteen, it will be absolutely necessary for the architect to provide a room where they can prepare their home tasks in peace and quiet. The parlour also acts as a reception-room, a room with fragrant and hallowed associations. One woman mentions that it is invaluable for an invalid or convalescent person. On the whole, it seems as if the case for the parlour is too strong to be overlooked.

